# In Vitro Evaluation of Ruminal Digestibility, Fermentation Characteristics, and Bacterial Diversity of Kenaf Crop at Various Cutting Heights

**DOI:** 10.3390/vetsci12010050

**Published:** 2025-01-12

**Authors:** Mengwei Li, Faiz-ul Hassan, Qian Lin, Muhammad Adeel Arshad, Muhammad Uzair Akhtar, Lijuan Peng, Chengjian Yang, Xin Liang, Jiaxiang Huang

**Affiliations:** 1 Guangxi Key Laboratory of Buffalo Genetics, Reproduction and Breeding, Guangxi Buffalo Research Institute, Nanning 530001, China; lmw1607@163.com (M.L.); lijuanpeng2000@163.com (L.P.); ycj0746@sina.com (C.Y.); liangxinbri@163.com (X.L.); 2 Faculty of Animal Production and Technology, Cholistan University of Veterinary and Animal Sciences, Bahawalpur 63100, Pakistan; f.hassan@cuvas.edu.pk (F.-u.H.); uzairakhtar@cuvas.edu.pk (M.U.A.); 3 Institute of Bast Fiber Crops, Chinese Academy of Agricultural Sciences, Changsha 410205, China; linqian@caas.cn; 4 Laboratory of Gastrointestinal Microbiology, National Center for International Research on Animal Gut Nutrition, Nanjing Agricultural University, Nanjing 210095, China; adeel.2203@gmail.com

**Keywords:** kenaf, forage, digestibility, fermentation characteristics, methane, bacterial diversity

## Abstract

The current study investigated the in vitro degradability, in vitro gas production, methane (CH_4_) production, and ruminal bacterial community of kenaf plants cut at different heights (130, 160, 190, 220, and 250 cm). These samples were subjected to an in vitro batch culture system using buffalo rumen fluid to measure gas and CH_4_ production at 3, 6, 9, 12, 24, 36, 48, and 72 h of incubation. Results reveal that crude protein (CP) concentration was the highest at the 220 cm height compared with the other heights. With the increase in height, gas and CH_4_ production decreased. However, the CH_4_ production at 190 cm was higher compared with the other plant heights. Dry matter degradation was higher at 190 cm and 220 cm, while ammonia-N and microbial CP were higher at the 220 cm height compared with the other heights. However, neutral detergent fiber degradation was the highest at the 130 cm height. Total volatile fatty acids, acetic acid, acetic acid/propane ratio, and pH value did not differ among the treatments, except for propionic acid, which was higher at the 130 cm and 160 cm heights. Overall, harvesting kenaf at plant heights of up to 220 cm was better in terms of its promising nutritional quality, improved dry matter degradation, and microbial CP contents.

## Simple Summary

Different alternate feed resources are being used to support sustainable livestock production and efficiently utilize the available resources. Kenaf is an industrial bast fiber crop that can be used as fodder or stored in the form of silage due to its excellent nutritional profile and environmental adaptations for growth. However, limited information on the cutting height and stage of maturity to use this crop as fodder causes a barrier for livestock producers to use it effectively as a feed ingredient. To identify the ideal plant height for maximum nutritional benefits, the current study was planned to examine the in vitro degradability, total gas and methane (CH_4_) production, and bacterial diversity of kenaf plants sampled at different heights (130, 160, 190, 220, and 250 cm). Results show that kenaf cut at a height of less than 250 cm has promising nutritional quality, in addition to dry matter digestibility and in vitro microbial crude protein content. We concluded that the use of kenaf in the early stage, before approaching the height of 250 cm, is the best to achieve optimum results.

## 1. Introduction

Improved production potential of modern livestock is primarily dependent upon the availability of quality feed [1]. The identification, characterization, and utilization of novel and alternate feed resources are becoming essential in this aspect. Various crop residues and byproducts are being used as alternative feeding sources without compromising the productivity of animals [2,3]. This includes several fiber crops, like ramie, jute, bamboo, napier grass, and kenaf [4]. Kenaf (*Hibiscus cannabinus* L.) is one of the most important herbaceous bast fiber crops, with excellent environmental adaptation characteristics, like high drought resistance, tolerance to salinity, wide adaptability [5], fast growth [6], and lower water requirements than maize and alfalfa [7]. Traditionally, it is cultivated in China, India, the USA, Malaysia, Indonesia, Bangladesh, Thailand, Vietnam, and South Africa [8,9]. It is of great interest to explore the nutritional value of kenaf to support the sustainable livestock production.

Kenaf has a strong potential to be used as an alternative forage source alongside conventional forages. It can be offered either as a fresh forage or preserved as silage for later utilization as a good feeding source for ruminants [10]. Kenaf silage has a better concentration of crude protein (CP), ether extract, and non-structural carbohydrates (6.6, 3.8, and 22% on DM basis, respectively) compared with ryegrass and rice straw silage. However, sometimes, the high moisture content in kenaf plants can cause difficulty in silage making [11]. Generally, kenaf has lower amounts of neutral detergent fiber (NDF) and acid detergent fiber (ADF) compared with other roughages such as rice straw and ryegrass [10]. As structural components of the plant, high NDF and ADF levels in feed result in reduced microbial protein synthesis, rumen volatile fatty acid (VFA) production, and milk yield in cows, as reported by Shi et al. [12]. Kenaf hay can partially replace alfalfa hay in the diet of finishing lambs with the same feed efficiency, indicating digestibility comparable to alfalfa hay [13,14]. Kenaf leaves and stem have 19.4 and 9.02% CP, respectively, during the immature growing stage (6 weeks), which later decline with plant maturity [15]. Despite the rich nutritive value of kenaf, various factors, such as growth stages, plant parts, and cultivar types, can affect its nutritional quality. For example, Ammar et al. [16] observed that CP content in kenaf leaves decreased with the advancing age of the plant and in vitro dry matter (DM) degradability and digestibility were also low in the later stages of harvesting. Differences in harvesting times and growth stages may also influence ruminal pH, methane (CH_4_) production, and ruminal microbial population [17]. A decrease in nutritional value and feed intake of kenaf silage harvested in the bloom stage compared with kenaf and dried beet pulp silage was also reported by Xiccato et al. [11]. Therefore, it is important to determine the nutritional value in different growth stages and measure the optimum height of kenaf plants before blooming, as Xiccato [18] mentioned that the apical part of kenaf shows better nutritional characteristics. Plant height is directly related to the growth and maturity of the plant, so it is imperative to test whether different plant heights at the same age may affect nutrient composition and digestibility. However, currently, limited information is available in terms of both the ideal harvesting height of the kenaf crop to be used as a forage and the effects of feeding kenaf to ruminants. Therefore, this study aimed to investigate (1) the nutrient composition of kenaf harvested at different heights and (2) the effects of kenaf harvested at different heights on rumen fermentation parameters, including the production of VFAs, ammonia, microbial protein synthesis, total gas, CH_4_, pH, nutrient digestibility, and changes in ruminal microbial community, before using this plant as a potential feed resource.

## 2. Materials and Methods

### 2.1. Planting and Harvesting of Kenaf

Kenaf was planted on the grassland of the Guangxi Buffalo Research Institute in April (temperature of 23.6 °C and relative humidity of 78.3%) by using the propagation method that uses vegetative organs to make independent plants. The actual average temperature during the growing phase was 29.1 °C, and the relative humidity was 81.9%. The total planting area was 8000 m^2^, and the planting land was hilly terrain with average annual rainfall of 1304.2 mm. Seeding was performed by adopting a ridge distance of 65 cm with double rows on the ridge. Seeding was 4–5 cm apart, and 5.2 kg urea, 11 kg diammonium, and 13.8 kg potassium sulfate were applied for every 667 m^2^. After every 50 days, timely watering with 9.5 kg urea and 9.2 kg potassium sulfate was applied for 667 m^2^. On an average, the total production of fresh biomass was 2.6 tons per 667 m^2^. Twenty kenaf plants with uniform heights for each height level were harvested at 130, 160, 190, 220, and 250 cm heights from the same field. Plants in all the treatment groups were sown at the same time in the month of April and harvested at the same time in the month of September. Plants were sampled at the 5th month of age. Therefore, harvested plants of all treatment groups were similar in age. Whole plants 5 cm above the ground were collected, crushed into 1–2 cm pieces by a small cutting machine, and dried at 65 °C for 72 h for further laboratory analysis.

### 2.2. Collection of Rumen Fluid and Preparation of Culture Media

Rumen fluid was collected from 3 healthy adult buffaloes equipped with permanent rumen fistulas reared at the Guangxi Buffalo Research Institute. Buffaloes were fed 8 kg of DM per day offered half at 08:00 and the remaining half at 15:00 h every day. Ingredient and nutrient composition of the diet is presented in Table 1. Animals had free access to fresh drinking water around the clock. Before morning feeding (07:30–08:00 h), animals were properly restrained, the fistula lid was opened, and rumen fluid was collected from different parts of the rumen, stored in carbon dioxide-filled thermos, and quickly brought back to the laboratory while maintaining anaerobic conditions.

The culture medium was prepared as previously reported by Theodorou et al. [19]. The composition of the fermentation broth is shown in Table 2. The culture medium was composed of 5 parts: A, B, C, D, and E. Among them, D was resazurin solution, which serves as an anaerobic indicator, exhibiting red coloration in the presence of oxygen and colorless under anaerobic conditions. A culture medium was prepared by adding 0.1, 200, 200, and 1 mL of solutions A, B, C, and D, respectively, into 558.9 mL of distilled water. After CO_2_ was saturated, it was retained in a water bath at a constant temperature of 39 °C for 5–6 h, 40 mL of solution E was added, and after mixing, CO_2_ was passed through until saturated, heated to 39 °C, and maintained thus for 0.5–1.0 h. A peristaltic pump separator was used to add 40 mL of buffer into a fermentation bottle (containing 1 g of fermentation substrate, with 6 replicates per treatment), and the whole process was permeated with CO_2_ and covered to seal the bottle. The fermentation bottle was preheated with buffer in a water bath to 39 °C for one hour before mixing with the rumen fluid. The rumen contents were filtered by four layers of gauze under CO_2_ conditions. A sterile syringe was used to inoculate 10 mL of rumen fluid into fermentation bottles before incubation at 39 °C for 72 h.

### 2.3. Determination of Nutrient Composition and Fermentation Characteristics

#### 2.3.1. Routine Nutrition Analysis

The samples were dried to pass a 100-mesh screen (Pulverisette 15, Fritsch Pulveris Ette, Idar-Oberstein, Germany) before further analysis. The contents of DM (method 934.01), organic matter (OM) through ash content (method 942.05), and CP (method 984.13) were measured following AOAC [20]. The NDF and ADF analyses were determined by following Van Soest et al. [21] with the ANKOM-2000 fiber analyzer (ANKOM Tech. Corp., Macedon, NY, USA). Energy contents were estimated by using a bomb calorimeter (PARR-6400 calorimeter; Moline, IL, USA).

#### 2.3.2. Determination of Fermentation Index and Nutrient Digestibility

During the fermentation process, dynamic gas production and CH_4_ production were measured for 3, 6, 9, 12, 24, 36, 48, and 72 h. The nonlinear model (NLIN) of MATLAB R2024a was used to fit the total gas production according to Zheng et al. [22]. With this method, the asymptotic gas produced (A; mL/g of DM), the sharpness defining the curve shape (B), the time required to reach half of the A (C), and the time of total in vitro incubation (t) were used to calculate the cumulative gas produced (GP_t_; mL/g of DM) at the t (h) incubation time by using the following formula:

GP_t_ = A/[1 + (C/t)^B^].

The rate of maximum substrate degradation (RmaxS, h) is [B × TRmaxS^(B − 1)^]/[C^B^ + TRmaxS^B^], the time at which the maximum rate of substrate degeneration (TRmaxS, h) is achieved is [C × (B − 1)^(1/B)^], the maximum rate of gas production (RmaxG, mL/h) is [A × C^B^ × B TRmaxG^(−B − 1)^]/[1 + C^B^ × TRmaxG^(−B)^]^2^, and the time at which RmaxG (h) is achieved is [C × {B − 1}/{B + 1}]^(1/B)^, as reported previously by Zheng et al. [22]. At each time node, a graduated glass syringe was used to balance the pressure inside and outside the fermentation bottle and record the volume of gas produced, the gas in the glass syringe was injected into the gas collection bag, and the methane concentration was detected by gas chromatography. Fermentation was terminated at 72 h by immediately placing the fermentation bottles on ice cubes, and the pH of the fermentation broth, VFAs, ammonia nitrogen (NH_3_-N), and the concentration of microbial protein (MCP) were determined following the methods presented in a previous study by Ebeid et al. [23]. pH was measured by using a pH meter (HANNA HI8424; Padova, Italy). For the measurement of NH_3_-N concentration, the phenol-hypochlorite reaction method was used following Xiccato [18]. The VFA concentrations, including acetic acid, butyric acid, and propionic acid, were estimated with an HP-INNOWAX (1909N-133, Agilent Technologies, Santa Clara, CA, USA) capillary column (30 m × 0.25 mm × 0.25 um), an inlet temperature of 200 °C, and crotonic acid as an internal standard by using a GC system (Agilent 7890A; Santa Clara, CA, USA) as previously reported by Qin [24].

Dry matter degradability (DMD), OM degradability (OMD), NDF degradability (NDFD), and ADF degradability (ADFD) were also calculated. For these degradability measurements, the remaining liquid with residues in the incubation bottles was filtered with nylon bags, which were already dried and weighed. After that, the remaining residues were completely washed with distilled water. After washing, the residues and bags were dried at 105 °C until they weighed constant as stated in a previous study by Guo et al. [25] for nutrient degradability calculations as described below:

Nutrient degradability = (1 − residue weight after digestion ÷ substrate weight before digestion) × 100

### 2.4. Determination of Rumen Bacteria Through 16-S RNA Gene Sequencing

At the end of the fermentation process, filtrate was used to extract the DNA as described previously by Hassan et al. [2]. Barcoded primers for the V3-V4 region were used for high-throughput 16S rRNA gene sequencing through the Illumina MiSeq PE300 platform (Illumina, San Diego, CA, USA). Raw sequence data were subjected to quality control, and assignment to operational taxonomic units (OTU) was performed as described in a previous study by Ebeid et al. [23]. Species annotation and the analysis of relative abundance were estimated by using the composition of bacterial communities in the rumen. Moreover, the differences among the treatment groups were elucidated by performing alpha diversity analysis.

### 2.5. Statistical Analysis

The data collected in this study were analyzed by using SPSS software (version 19.0) by the analysis of variance (ANOVA) technique. Duncan’s multiple range test was used to detect significant differences among the treatment groups. The results were declared significant at *p* < 0.05. R software (version 3.3.1) was used to conduct the principal component analysis (PCA) of all groups for the beta diversity index. The composition of different sample communities can be analyzed to reflect the difference and distance between the samples. PCA uses variance decomposition to reflect the differences among multiple groups of data on a two-dimensional coordinate map, and the coordinate axes are selected to reflect the two characteristic values that can best reflect the differences among samples.

## 3. Results

### 3.1. Nutrient Composition of Kenaf at Different Heights

The nutrient composition of kenaf plants harvested at different heights is presented in Table 3. The DM, OM, and ash contents were similar among the different height groups (*p* > 0.05). The highest CP and energy contents were observed in plants harvested at the 220 cm height compared with the others (*p* < 0.05). The NDF and ADF contents were highest in kenaf plants cut at the 250 and 160 cm heights compared with those cut at the heights of 130, 190, and 220 cm (*p* < 0.05).

### 3.2. In Vitro Gas and CH_4_ Production

**Figure 1:**
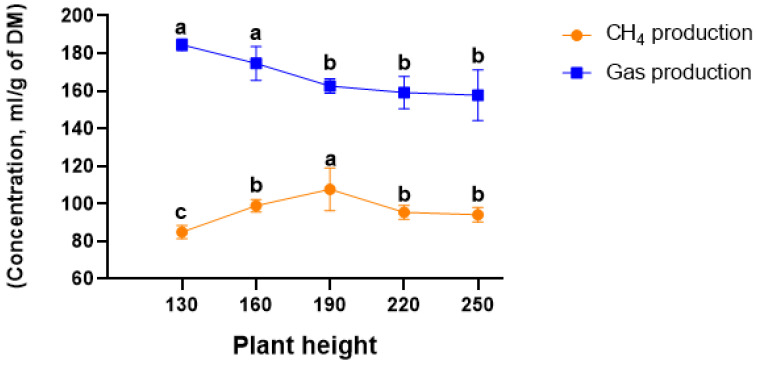
Total gas and CH_4_ production of kenaf plants of different heights (values with different superscripts in the figure differ significantly (*p* < 0.05)).

The effects of plant height on in vitro total gas and methane production are presented in Figure 1. The highest gas production was observed at 130 and 160 cm compared with other groups (*p* < 0.05). CH_4_ production was the highest at 190 cm and the lowest at 130 cm compared with the other height groups (*p* < 0.05).

The relations of methane production with total gas production and in vitro nutrient digestibility are presented in Table 4. A positive correlation between methane and gas production was detected, while CH_4_ production was negatively correlated with DNDF. A positive correlation between gas production and DNDF was also observed.

The effects of kenaf harvested at different heights on total gas production and in vitro rumen fermentation kinetics are presented in Table 5. The highest values of GP_72_ (mL/g DM) were observed with the plant heights of 130 and 160 cm compared with the other heights (*p* < 0.05). A decrease in A (mL) and RmaxG (mL/h) was also noted with a plant height of 190 cm and above, compared with the plants cut at lower heights (*p* < 0.05). Different harvesting heights of kenaf had similar B (h), C (h), TRmaxG (h), TRmaxS (h), and RmaxS (mL/h; *p* > 0.05).

### 3.3. In Vitro Nutrient Degradability

The nutrient degradability of kenaf at different heights is presented in Table 6. The DMD of kenaf harvested at 190 and 220 cm was higher compared with the plants cut at relatively higher or lower heights (*p* < 0.05). The highest OMD among different groups was observed at the cutting height of 160 cm, while the lowest OMD was observed at the plant height of 250 cm (*p* < 0.05). The harvesting height of 130 cm had the highest NDFD, while 220 cm had the lowest values (*p* < 0.05). ADFD was higher at the 160 and 190 cm heights compared with 220 cm (*p* < 0.05).

### 3.4. In Vitro Rumen Fermentations

The effects of kenaf harvested at different heights on the in vitro rumen fermentation parameters are presented in Table 7. Total VFA concentration, acetic acid, acetic acid-to-propionic acid ratio, and pH value did not differ among different groups (*p* > 0.05). The concentration of propionic acid was higher at 130 and 160 cm compared with the 200 cm cutting height (*p* < 0.05). The lowest butyric acid concentration was observed at the plant height of 220 cm compared with the other groups (*p* < 0.05). The ammonia-N and MCP contents were the highest at the 220 cm kenaf height compared with the others (*p* < 0.05).

### 3.5. Rumen Bacterial Diversity

**Figure 2:**
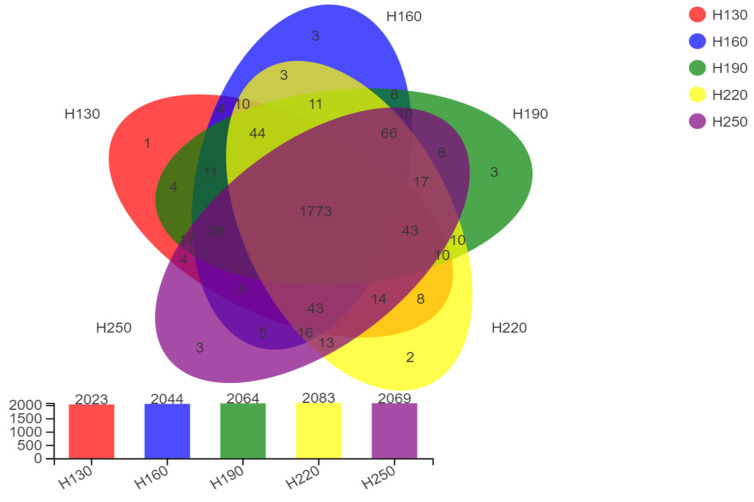
Venn diagram depicting unique and shared OTUs among treatments (H130: 130 cm; H160: 160 cm; H190: 190 cm; H220).

In total, 10,283 OTUs in five treatments having different plant heights were detected by high-throughput 16S rRNA gene sequencing. The unique and shared OTU distribution of all treatments is presented in Figure 2. An improvement in the number of OTUs was observed in response to the increase in the harvesting height of kenaf, up to the height of 220 cm. All groups shared 1773 OUTs in total, whereas 12 unique OUTs were detected.

**Figure 3:**
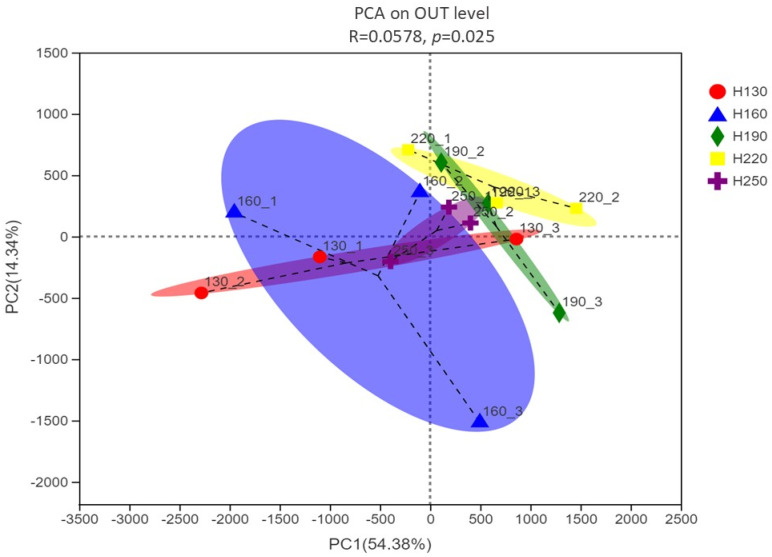
Venn diagram depicting unique and shared OTUs among treatments (H130: 130 cm; H160: 160 cm; H190: 190 cm; H220: 220 cm; H250: 250 cm).

The alpha diversity parameters of rumen bacteria among different treatment groups are presented in Table 8. Sobs, shannon, simpson, ACE, chao, coverage, shannoneven, and simpsoneven were similar among different harvesting heights of kenaf (*p* > 0.05). The ACE index was greater for the plants harvested at 220 cm compared with the others (*p* < 0.05). The first two components of PCA presented in Figure 3 are PC1 and PC2, which explain 54.38 and 14.34% of the total variance, respectively. The variability of the replicates within groups is shown by using ellipses and displayed a decrease in the variability of microbial communities in the 250 cm height group. The largest variability was found in the 160 height group.

### 3.6. Relative Abundance of Bacterial Phyla

**Figure 4:**
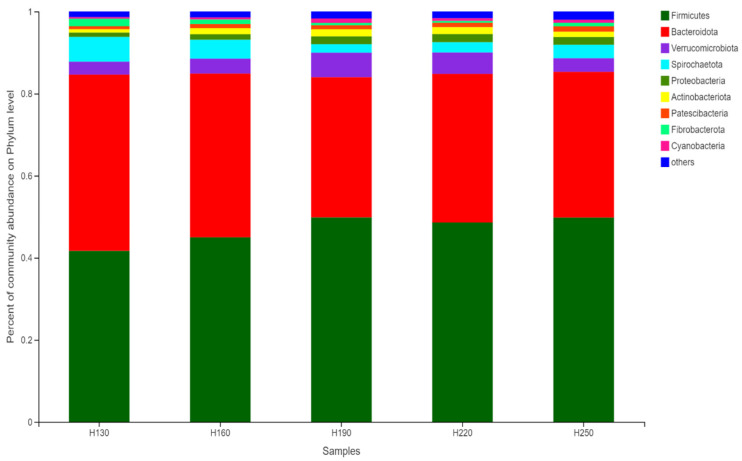
The relative abundance of different bacterial communities among the treatment groups.

The relative abundance of bacterial phyla is presented in Figure 4. The relative abundance of various bacterial phyla indicated that Firmicutes (41.83–50.21%) and Bacteroidetes (35.35–42.86%) were the most dominant phyla detected in all treatments. The remaining phyla were Verrucomicrobiota (3.13–5.81%), Spirochaetota (2.06–6.02%), Proteobacteria (1.07–1.99%), and some others. However, the relative abundance of bacterial phyla was not different among different height groups (*p* > 0.05).

### 3.7. Relative Abundance at Genus Level of Different Bacteria

**Figure 5:**
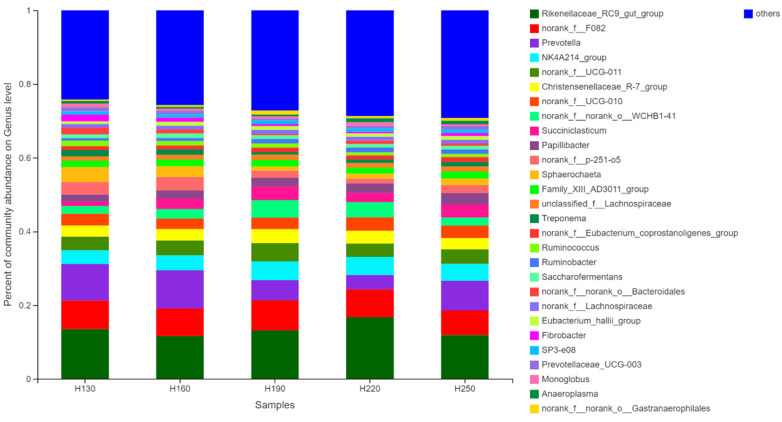
The relative abundance at the genus level of different bacteria among the study groups (H130: 130 cm; H160: 160 cm; H190: 190 cm; H220: 220 cm; H250: 250 cm).

The relative abundance of different bacteria at the genus level in response to plant height is presented in Figure 5. In total, more than 30 bacterial genera were identified, including the 12 major genera: Rikenellaceae_RC9_gut_group (9.5–15.25%), Prevotella (3.99–10.97%), norank_f__F082 (6.65–8.11%), Ruminococcaceae_NK4A214_group (3.70–5.12%), norank_f__UCG-011 (3.64–4.99%), Christensenellaceae_R-7_group (3.02–3.86%), norank_f__UCG-010 (2.81–3.60%), norank_f__norank_o__WCHB1-41 (2.11–4.62%), Succiniclasticum (1.18–3.73%), norank_f__p-251-o5 (1.29–3.55%), Papillibacter (1.90–3.09%), and Sphaerochaeta (1.12–4.08%). However, there was no significant difference among the study groups (*p* > 0.05).

## 4. Discussion

The variations in kenaf in vitro gas generation, CH_4_ production, and other fermentation characteristics caused by plant height reflect different nutritional component levels in the experimental samples. The nutrient contents in kenaf observed in this study are in agreement with previous studies, while some differences in studies regarding the nutrient composition of kenaf can be attributed to harvesting stage, soil conditions, fertilizers used, crop varieties, and other environment and management conditions [4,26,27,28]. Plant height generally affects not only the chemical composition but also the nutritional value for animals [29]. Moreover, plant height is considered to be negatively related with the CP content, as taller plants have low CP and high fiber contents compared with short plants [30]. Therefore, in the current study, an increase in CP up to 220 cm height and then a decrease with a further increase in plant height indicated the height limits for the harvesting of kenaf. It is reported that plant height effects the in vitro rumen fermentation kinetics and nutritional values [31,32]. The increase in the height of the kenaf plant in our study resulted in decreased total gas production, which is consistent with the findings reported by Guo et al. [27]. Lower gas volume could be attributed to the decrease in nutritional value and vice versa [33]. However, as plant height increased, gas production decreased, which is consistent with Wilman et al. [34]. Lower CH_4_ production was observed at the 130 cm height, which may be due to the low CP content of that group. For instance, in a diet with decreased CP content, bacteria (*Prevotella* and *Butyrivibrio*) need a longer time to decompose nitrogen compounds to produce the raw materials needed for the reproduction and synthesis of methanogenic archaea and/or CH_4_ synthesis [35]. During the CH_4_ formation process, hydrogen, acting as a precursor, is accompanied by acetic acid [36]. Meanwhile, intermediates of the citric acid cycle, like fumaric acid and malic acid, can use the hydrogen to produce the propionate. Consequently, this competition for hydrogen affects CH_4_ production [37]. During the early stage, more soluble carbohydrates may enhance the production of propionate in the rumen with a reduction in CH_4_ production due to the inhibition of methanogen growth [38]. The lower CH_4_ of high-quality forage was due to shifting fermentation towards more proponent production [39]. Lower IVDMD at 160 and 250 cm and OMD at the 190 and 250 cm heights is likely due to the high amount of fiber contents (NDF and ADF), as reported previously [40,41,42]. Lower NDFD is also likely related to increased fiber content with the increase in plant height [33]. Generally, digestibility is reported to be dependent on cell-wall (i.e., NDF) content, which changes during the development or until reaching a particular stage of maturity [43]. However, the tallest stem may not be the most mature stem [44]. The shoots and leaves of plants are harvested before secondary wall formation, which decreases digestibility by acetylation and/or lignification [45]. The plants were growing, and a height up of to 190 or 220 cm might be the maximum height of kenaf growth without reaching that secondary wall formation stage. In addition, plant height had no effect on pH, acetate content, acetate-to-propionate ratio, and total VFA content during in vitro fermentation. VFAs are the end products of ruminal fermentation and contribute 50–70% of total metabolizable energy in ruminants [2]. Butyrate concentration was increased with plant height. Our findings are consistent with the findings of Haque [46] that increasing fiber content in the diet could result in higher butyrate synthesis compared with a concentrate-rich diet. The greater concentration of propionate at the heights of 130 and 160 cm can be attributed to the greater proportion of non-structural carbohydrates due to early maturity. The average amounts of NH_3_-N and MCP produced at the 220 cm plant height were higher than the other ones. The results show that nutrient contents and proportion might lead to different interactions during fermentation and the synthesis efficiency of MCP at the height of 220 cm.

In ruminants, ruminal microorganisms have a key role in the conversion of fiber into digestible compounds. In this study, 16-S RNA sequencing was performed to investigate the diversity in ruminal bacterial. Bacterial diversity remained unaffected by plant height in this study. However, the ACE index was greater in the 220 cm height group compared with the others. Firmicutes and Bacteroidetes were the dominant phyla among all groups, which agreed with the results of previous studies that Firmicutes and Bacteroidetes are numerically the most dominant phyla in the microbiome of ruminants [23,47,48]. The phylum Bacteroidetes can hydrolyze complex OM macromolecules and subsequently utilize their small units to produce acetate, lactate, and succinate [49,50]. Firmicutes are associated with the production various extracellular enzymes and all types of proteases, lipases, and cellulases, which ultimately lead to the hydrolysis of complex macromolecules like protein, lipids, carbohydrates, and fiber [51]. Similar to the bacterial phyla, the most dominant genera among all groups were *Prevotella* and *Rikenellaceae-RC9*. *Prevotella*, the most abundant genus in cow rumen is reported to be associated with carbohydrate and protein digestion in the rumen [52]. *Prevotella* is a genus with several roles, the most important of which includes increasing the degradation of proteins and aiding other strains in improving the utilization of fiber resources in ruminants [53]. It is also observed that colonization trends are different for bacterial species to attach to various parts of plants according to the surface chemistry and their requirements for growth and survival [54]. Keeping in view all these aspects, the results might be different for plants cut at different ages and heights than used in this study. This study will be helpful in better understanding the harvesting height of the kenaf crop to achieve maximum benefits in terms of nutritional profile, digestibility, rumen fermentation, and microbial protein synthesis without compromising ruminal biodiversity. However, in vivo studies and kenaf processing methods are warranted to find and improve the optimum dietary inclusion level, observe the production responses, and define the interactions of kenaf with other sources for the efficient utilization of this industrial crop. Interestingly, the differences in ruminal microbiota of cow and buffalo may also influence nutrient digestibility, as buffalo has been reported to have lower dietary protein requirements with even better fiber degradation potential [55]. As per the authors’ knowledge, the present study is the first one reporting bacterial diversity in response to kenaf harvested at different heights and observed no negative effects of various plant heights on all ruminal genera. However, our findings contradict previous research using other dietary sources, where considerable changes in the bacterial ecology as a result of different harvest stages were observed, which can be explained by the fact that the surface chemistry of kenaf might be different from other sources and plant ages in our study groups were similar [56].

## 5. Conclusions

Overall, the difference in the harvesting height of kenaf affected the nutrient composition of the plants in this study. CP and energy contents increased up to the harvesting height of 220 cm and decreased with further increases in plant height. The digestibility of organic matter and NDF was decreased with the increase in plant height. Plant heights higher than 160 and below 250 cm are promising in terms of nutrient composition, dry matter digestibility, and microbial protein synthesis. Based on these results, it can be concluded that kenaf can be successfully used as a ruminant feed resource, but it is better to use it in the early stage, before approaching the height of 250 cm. These findings can be used not only by farmers for the ideal harvesting of kenaf to achieve maximum nutritional benefits for livestock but also by scientists for further understanding gas production, methane production, and bacterial diversity in response to the utilization of this industrial crop as a feed resource and exploring other options to further improve outcomes.

## Figures and Tables

**Table 1:** Ingredient and nutrient composition of the diet (dry matter basis).

Ingredient	% of DM
Elephant grass	90.0
Corn	4.80
Wheat bran	1.20
Soybean meal	1.80
Cottonseed meal	1.00
Rapeseed meal	0.60
Lime stone	0.10
CaHPO_4_	0.10
NaHCO_3_	0.15
NaCl	0.15
Premix	0.10
Nutrient composition	
Crude protein (% of DM)	8.58
Neutral detergent fiber (% of DM)	61.7
Acid detergent fiber (% of DM)	38.1
Crude ash (% of DM)	6.31
Energy (KJ/g)	15.5

**Table 2:** Composition of fermentation buffer.

S. No.	Solution	Volume (mL)	Composition
A	Trace elements	100	CaCl_2_·2H_2_O 13.2 g, MnCl_2_·4H_2_O 10.0 g, CoCl_2_·6H_2_O 1.0 g, FeCl_3_·6H_2_O 8.0 g
B	Buffer	1000	NH_4_HCO_3_ 4.0 g, NaHCO_3_ 35.0 g
C	Constant element	1000	Na_2_HPO_4_ 5.7 g, KH_2_PO_4_ 6.2 g, MgSO_4_·7H_2_O 0.6 g
D	Resazurin	1	0.10%
E	Reducing agent	100	L-Cys·HCl 0.625 g, NaOH 0.16 g, Na_2_S·9H_2_O 0.625 g

**Table 3:** Nutrient composition of kenaf of different heights (dry matter basis).

Height	DM (%)	OM (%)	Ash (%)	CP (%)	Energy (KJ/g)	NDF (%)	ADF (%)
250 cm	29.9	25.9	3.99	8.61 ^c^	17.51 ^bc^	46.9 ^a^	31.8 ^a^
220 cm	29.3	25.4	3.93	11.9 ^a^	18.10 ^a^	42.3 ^c^	26.9 ^c^
190 cm	29.1	24.8	4.26	10.1 ^b^	17.38 ^c^	44.4 ^b^	30.0 ^b^
160 cm	30.9	27.1	3.82	8.15 ^c^	17.26 ^c^	47.3 ^a^	32.2 ^a^
130 cm	30.2	26.1	4.09	8.07 ^c^	17.81 ^ab^	43.2 ^c^	30.1 ^b^
SEM	0.473	0.470	0.303	0.259	0.127	0.300	0.438
*p*-Value	0.126	0.056	0.869	<0.001	0.005	<0.001	<0.001

DM: dry matter; OM: organic matter; CP: crude protein; NDF: neutral detergent fiber; ADF: acid detergent fiber. SEM: standard error of the mean. Values in the same column with different superscript differ significantly (*p* < 0.05).

**Table 4:** Methane production in relation to total gas production and digestibility.

Items	CH_4_ Production	Gas Production	DMD	OMD	NDFD	ADFD
CH_4_ production	1					
Gas production	−0.417 *	1				
DMD	0.165	0.060	1			
OMD	−0.338	0.259	−0.458 *	1		
NDFD	−0.435 *	0.726 **	0.101	0.143	1	
ADFD	0.230	0.216	−0.259	0.001	0.340	1

* *p* < 0.05 and ** *p* < 0.01.

**Table 5:** Effects of different heights on gas production and kinetic parameters.

Parameter	Plant Height					SEM	*p*-Value
	130 cm	160 cm	190 cm	220 cm	250 cm		
GP_72_ (mL/g DM)	184.5 ^a^	174.7 ^a^	162.7 ^b^	159.2 ^b^	157.7 ^b^	3.466	<0.001
A (mL)	206.7 ^a^	194.9 ^b^	182.6 ^c^	180.5 ^c^	181.1 ^c^	3.706	<0.001
B (h)	1.45	1.52	1.53	1.52	1.55	0.032	0.270
C (h)	12.80	13.00	13.27	13.12	13.48	0.360	0.731
TRmaxG (h)	4.03	4.64	4.76	4.57	5.05	0.256	0.108
RmaxG (mL/h)	9.90 ^a^	9.18 ^b^	8.41 ^c^	8.46 ^c^	8.20 ^c^	0.247	<0.001
TRmaxS (h)	7.47	8.51	8.73	8.38	9.23	0.435	0.102
RmaxS (mL/h)	6.12	6.18	6.07	6.19	6.04	0.187	0.970

SEM = standard error of the mean. Values with different superscripts in the same row differ significantly (*p* < 0.05).

**Table 6:** Nutrient degradability of kenaf plants at different heights.

Parameter	Plant Height					SEM	*p*-Value
	130 cm	160 cm	190 cm	220 cm	250 cm		
DMD (%)	60.5 ^b^	58.4 ^c^	62.3 ^a^	61.0 ^ab^	58.5 ^c^	0.565	<0.001
OMD (%)	74.1 ^b^	80.2 ^a^	63.4 ^d^	76.0 ^b^	70.6 ^c^	0.924	<0.001
NDFD (%)	57.5 ^a^	54.0 ^b^	52.4 ^bc^	49.9 ^c^	51.3 ^bc^	0.825	0.001
ADFD (%)	48.1 ^ab^	50.6 ^a^	49.4 ^a^	44.6 ^b^	48.2 ^ab^	1.095	0.029

SEM = standard error of the mean. Values with different superscripts in the same row differ significantly (*p* < 0.05).

**Table 7:** In vitro rumen fermentation of kenaf plants at different heights.

Parameter	Plant Height					SEM	*p*-Value
	130 cm	160 cm	190 cm	220 cm	250 cm		
Acetic acid (mmol/L)	19.2	19.3	20.1	19.0	19.9	0.881	0.874
Propionic acid (mmol/L)	9.71 ^a^	9.16 ^a^	8.93 ^ab^	8.00 ^b^	8.87 ^ab^	0.318	0.040
Acetic acid/Propionic acid	1.97	2.10	2.25	2.38	2.24	0.123	0.245
Butyric acid (mmol/L)	4.17 ^ab^	4.71 ^a^	4.50 ^a^	3.59 ^b^	4.45 ^a^	0.222	0.039
Total VFAs (mmol/L)	33.1	33.2	33.6	30.6	33.2	1.129	0.385
pH	6.13	6.18	6.12	6.16	6.23	0.031	0.190
NH_3_ (mg/100 mL)	5.77 ^b^	5.56 ^b^	6.15 ^b^	7.26 ^a^	5.67 ^b^	0.203	0.001
MCP (mg/mL)	20.7 ^ab^	18.6 ^b^	19.9 ^b^	24.0 ^a^	19.9 ^b^	1.073	0.045

SEM: standard error of the means; VFAs: volatile fatty acids; MCP: microbial crude protein. Values in the same row with different superscripts differ significantly (*p* < 0.05).

**Table 8:** Alpha diversity parameters of rumen bacteria among all treatments.

Items	H130	H160	H190	H220	H250	SEM	*p*-Value
Sobs	1701	1702	1748	1788	1783	28.183	0.139
Shannon	5.98	5.99	6.03	6.08	6.14	0.047	0.151
Simpson	0.008	0.008	0.008	0.007	0.006	0.001	0.414
ACE	1934 ^b^	1941 ^b^	1978 ^ab^	2020 ^a^	1991 ^ab^	17.93	0.035
Chao	1953	1977	2010	2039	2014	19.13	0.067
Coverage	0.992	0.992	0.992	0.993	0.993	0.001	0.527
Shannoneven	0.804	0.806	0.808	0.813	0.821	0.005	0.229
Simpsoneven	0.074	0.073	0.076	0.086	0.088	0.007	0.485

SEM: standard error of the means. Values in the same row with different superscripts differ significantly (*p* < 0.05).

## Data Availability

The data are available upon reasonable request from the corresponding author.
